# Prediction of treatment response in patients with brain metastasis receiving stereotactic radiosurgery based on pre-treatment multimodal MRI radiomics and clinical risk factors: A machine learning model

**DOI:** 10.3389/fonc.2023.1114194

**Published:** 2023-03-13

**Authors:** Peng Du, Xiao Liu, Li Shen, Xuefan Wu, Jiawei Chen, Lang Chen, Aihong Cao, Daoying Geng

**Affiliations:** ^1^ The Second Affiliated Hospital of Xuzhou Medical University, Xuzhou, Jiangsu, China; ^2^ Huashan Hospital, Fudan University, Shanghai, China; ^3^ School of Computer and Information Technology, Beijing, China; ^4^ Department of Radiology, Jiahui International Hospital, Shanghai, China; ^5^ Department of Radiology, Shanghai Gamma Hospital, Shanghai, China

**Keywords:** stereotactic radiosurgery, brain metastasis, treatment response, multimodal MRI, radiomics, machine learning

## Abstract

**Objectives:**

Stereotactic radiosurgery (SRS), a therapy that uses radiation to treat brain tumors, has become a significant treatment procedure for patients with brain metastasis (BM). However, a proportion of patients have been found to be at risk of local failure (LF) after treatment. Hence, accurately identifying patients with LF risk after SRS treatment is critical to the development of successful treatment plans and the prognoses of patients. To accurately predict BM patients with the occurrence of LF after SRS therapy, we develop and validate a machine learning (ML) model based on pre-treatment multimodal magnetic resonance imaging (MRI) radiomics and clinical risk factors.

**Patients and methods:**

In this study, 337 BM patients were included (247, 60, and 30 in the training set, internal validation set, and external validation set, respectively). Four clinical features and 223 radiomics features were selected using least absolute shrinkage and selection operator (LASSO) and Max-Relevance and Min-Redundancy (mRMR) filters. We establish the ML model using the selected features and the support vector machine (SVM) classifier to predict the treatment response of BM patients to SRS therapy.

**Results:**

In the training set, the SVM classifier that uses a combination of clinical and radiomics features demonstrates outstanding discriminative performance (AUC=0.95, 95% CI: 0.93-0.97). Moreover, this model also achieves satisfactory results in the validation sets (AUC=0.95 in the internal validation set and AUC=0.93 in the external validation set), demonstrating excellent generalizability.

**Conclusions:**

This ML model enables a non-invasive prediction of the treatment response of BM patients receiving SRS therapy, which can in turn assist neurologist and radiation oncologists in the development of more precise and individualized treatment plans for BM patients.

## Introduction

Brain metastasis (BM) is one of the most common intracranial malignant tumors in adults, and more than 20% of cancer patients develop BM during the course of the disease (1). At present, the treatment methods of BM consist of surgery, radiotherapy, chemotherapy, targeted therapy, immunotherapy and comprehensive treatment, among which, radiotherapy is one of the most important treatment procedures, including the whole brain radiotherapy (WBRT) and the stereotactic radiosurgery (SRS) (2, 3). In recent years, SRS has been increasingly used in the treatment of patients with BM due to its efficacy, short treatment time and low neurotoxicity (4, 5). According to the guidelines of the American Society for Radiation Oncology (6), level-1 evidence shows exclusive SRS therapy is appropriate for patients with less than four metastases. Although the local control rate of SRS for BM can reach more than 70% (7), a proportion of patients still suffer from local failure (LF) after treatment (8, 9). The treatment response of BM is typically determined using the change in tumor size in structural brain MRI, which is evaluated manually by radiation oncologists. However, changes in the physical size of the tumor may take months to become identifiable in subsequent MRI images (10), which may lead to time delays in the appropriate adjustment of treatment plans. Therefore, if accurate prediction can be made in advance, the treatment response of BM after SRS therapy can effectively facilitate the development of personalized treatment plans, reduce complications and adverse reactions due to ineffective treatment, and improve the prognoses of patients.

Radiomics is a new discipline that combines traditional radiology, big data analysis and precision medicine. By extracting a large number of features from standardized radiological data and establishing predictive models after feature screening, it has been applied to clinical decision-making systems to assist diagnosis and evaluate prognosis, which has gained increasing importance in oncology research (11–14). Numerous studies have shown that the radiomics features of pre-treatment radiology images can be used to predict the treatment response of some diseases. Kickingerer et al. (15) extracted 4842 quantitative radiomics features from pre-treatment MRI images (T1 weighted imaging (T1WI), contrast enhanced-T1WI (CE-T1WI), and T2-fluid attenuated inversion recovery (T2-FLAIR) sequences) of 172 recurrent glioblastoma (r-GBM) patients, and established a model that could efficaciously predict the sensitivity of r-GBM patients to bevacizumab treatment. Liu et al. (16) developed a radiomics model containing radiomics features and independent clinicopathological risk factors to predict the pathological complete response of patients with locally advanced rectal cancer to neoadjuvant chemoradiotherapy, with an AUC of 0.9756 in the validation set. The research of Dercle et al. (17) indicated that radiomics features extracted from pre-therapy chest CT images could accurately predict the sensitivity of non-small cell lung cancer patients to chemotherapy and targeted therapy.

Currently, most studies of radiomics in BM have focused on the differentiation between true progression (TP) and radiation necrosis (RN) after radiotherapy (18–21). However, few investigations have been done on the prediction of treatment response of BM patients who have received SRS therapy, and in some contexts, this prediction possesses more clinical significance than the prediction of survival. Mouraviev et al. (18) retrospectively analyzed the 408 BM in 87 patients treated with SRS, and a total of 440 radiomic features were extracted from the tumor core and the peritumoral regions, using the baseline pretreatment post-contrast T1 and T2-FLAIR MRI sequences. They found that radiomic features aid local control prediction of BM treated with radiosurgery and radiomic features are complementary to clinical features for this task. Huang et al. (22) retrospectively analyzed the data of 161 patients with non-small cell lung cancer (576 BM) who underwent Gamma Knife Radiosurgery (GKRS) for BM, and it was indicated that the zone percentage of brain metastases, a radiomic feature derived from pre-GKRS contrast-enhanced T1-weighted MRIs, was found to be an independent prognostic factor of local tumor control following GKRS in patients with non-small cell lung cancer and brain metastases. While the SRS therapy has been shown to be effective in the improvement in the local control of BM, it does not always imply the improvement in the survival (23). The main significance of SRS therapy may still mainly lie in the alleviation of the relevant neurological symptoms and improvement of the quality of life (24–26). Avoiding overtreatment of patients with poor prognosis is as important as the active treatment of patients who are likely to survive for several years (27). Therefore, if the patients at risk of LF after receiving SRS therapy can be identified in the first place, this information can be essential for neuro-oncology physicians to create appropriate treatment plans, such as surgery or WBRT, to reduce the burden and adverse reactions due to less effective treatments, thereby ameliorating the treatment effect (3).

To develop accurate predictions of the treatment response of BM patients to SRS therapy, we propose a novel non-invasive SRS efficacy prediction model by employing an ML approach, combining the pre-treatment multimodal MRI radiomics features and relevant clinical risk factors. We show that the model is capable of producing accurate predictions of BM patients who are at risk of LF after SRS therapy.

## Patients and methods

### Patients

This retrospective study was approved by the Institutional Review Boards. All patients or their guardians have given their informed consent to the utilization of their anonymized MRI images and clinical data for research purposes. In total, 307 BM patients treated with SRS between January 2015 and November 2020 in Huashan Hospital, Fudan University were enrolled in the study. Among them, 247 and 60 patients were randomly assigned to the training and internal validation sets through a stratified sampling method. In addition, 30 BM patients treated with SRS between January 2015 and November 2020 in the Second Affiliated Hospital of Xuzhou Medical University were utilized for external validation. The demographic and clinical characteristics of the patients were shown in **Table 1**. Eligible criteria were as follows: (1) pathologically confirmed primary cancer; (2) no more than four brain metastases confirmed by CE-T1WI of brain MRI; (3) Karnofsky Performance Status (KPS)≥70; (4) patients who underwent SRS therapy only; (5) patients who didn’t receive surgery or WBRT before SRS therapy; (6) complete acquisition images of pre-therapy and follow-up MRI, including T1WI, CE-T1WI, T2 weighted imaging (T2WI) and diffusion weighted imaging (DWI); (7) complete relevant clinical data.

**Table 1:** Characteristics of patients and lesions in all datasets.

Variables	Training set	Internal validation set	External validation set
No. of patients	247	60	30
Age (years): median (range)	60 (22-78)	61 (37-76)	64 (46-74)
Female: Male	115:132	22:38	12:18
No. of metastases per patient: median (range)	1 (1-4)	1 (1-3)	1.5 (1-4)
Total number of metastases	403	101	55
KPS score: median (range)	80 (70-90)	70 (70-100)	70 (70-80)
GPA score: median (range)	2.5 (0.5-4)	2.5 (1-4)	2 (0.5-2.5)
Location of tumors: No. (%)			
Frontal lobe	180 (44.7%)	35 (34.7%)	12 (21.8%)
Occipital lobe	48 (11.9%)	12 (21.7%)	5 (9.1%)
Temporal lobe	42 (10.4%)	10 (16.7%)	9 (16.4%)
Parietal lobe	69 (17.1%)	18 (13.3%)	13 (23.6%)
Cerebellum	37 (9.2%)	15 (25.0%)	9 (16.4%)
Brainstem	5 (1.2%)	2 (3.3%)	2 (3.6%)
Others	22 (5.5%)	9 (15.0%)	5 (9.1%)
Primary tumor type: No. (%)			
Lung Cancer	186 (75.3%)	41 (68.3%)	23 (76.7%)
Breast Cancer	24 (9.7%)	8 (13.3%)	1 (3.3%)
Colorectal Cancer	13 (4.3%)	3 (5.0%)	3 (10.0%)
Kidney Cancer	5 (5.3%)	6 (10.0%)	0
Gynecologic Cancer	5 (5.3%)	0	0
Others	14 (5.7%)	2(3.3%)	3 (10.0%)
Tumor volume (cm^3^): median (range)	2.58 (0.39-51)	2.47 (0.51-37)	2.73 (0.45-42)
Edema index: median (range)	4.32 (1-57.6)	3.26 (1-53.7)	3.01 (1.53-26.7)

KPS, Karnofsky Performance Status; GPA, Graded Prognositic Assessment.

### Treatment response assessment

All patients underwent SRS therapy using Leksell Gamma Knife^Ⓡ^ Perfexion™ (Elekta, Norcross, GA, USA). The median radiosurgery volume was 3.16 cm^3^ and the median margin dose was 17 Gy (range 15–20 Gy). The margin dose was generally prescribed at an isodose line level of 40%–70% with a median of 50%. The median time between pre-treatment MRI and SRS therapy was 0 day (range, 0-4 days).

Patients were evaluated with MRI at the pre-treatment and the follow-up approximately 60 days after SRS therapy. Tumor response was estimated based on the Response Assessment in Neuro-Oncology Brain Metastases (RANO-BM) criteria (28), which classifies patients into complete response (CR), partial response (PR), stable disease (SD), and progressive disease (PD). We defined the CR and PR as locally effective (LE) group, the SD as locally stable (LS) group, and the PD as locally ineffective (LIE) group.

### Image acquisition

Axial CE-T1WI, T2WI and DWI images of all patients were acquired on 1.5T MRI system (SIGNA Excite HD; GE Healthcare, Milwaukee, WI, USA) with an 8-channel phased-array head coil. The scanning parameters were as follows: CE-T1WI [repetition time (TR) = 800ms, echo time (TE) = 7.7ms, bandwidth = 122Hz, slice thickness (ST) = 3mm, slice gap = 0mm]; T2WI (TR = 5500ms, TE = 97ms, bandwidth = 122Hz, ST = 3mm, slice gap = 0mm); DWI (TR = 4400ms, TE = 101ms, bandwidth = 1022Hz, ST = 6mm, slice gap = 2mm); field of view (FOV) = 240mm × 240mm; acquisition matrix = 256 × 256.

### MRI data analysis

**Figure 1:**
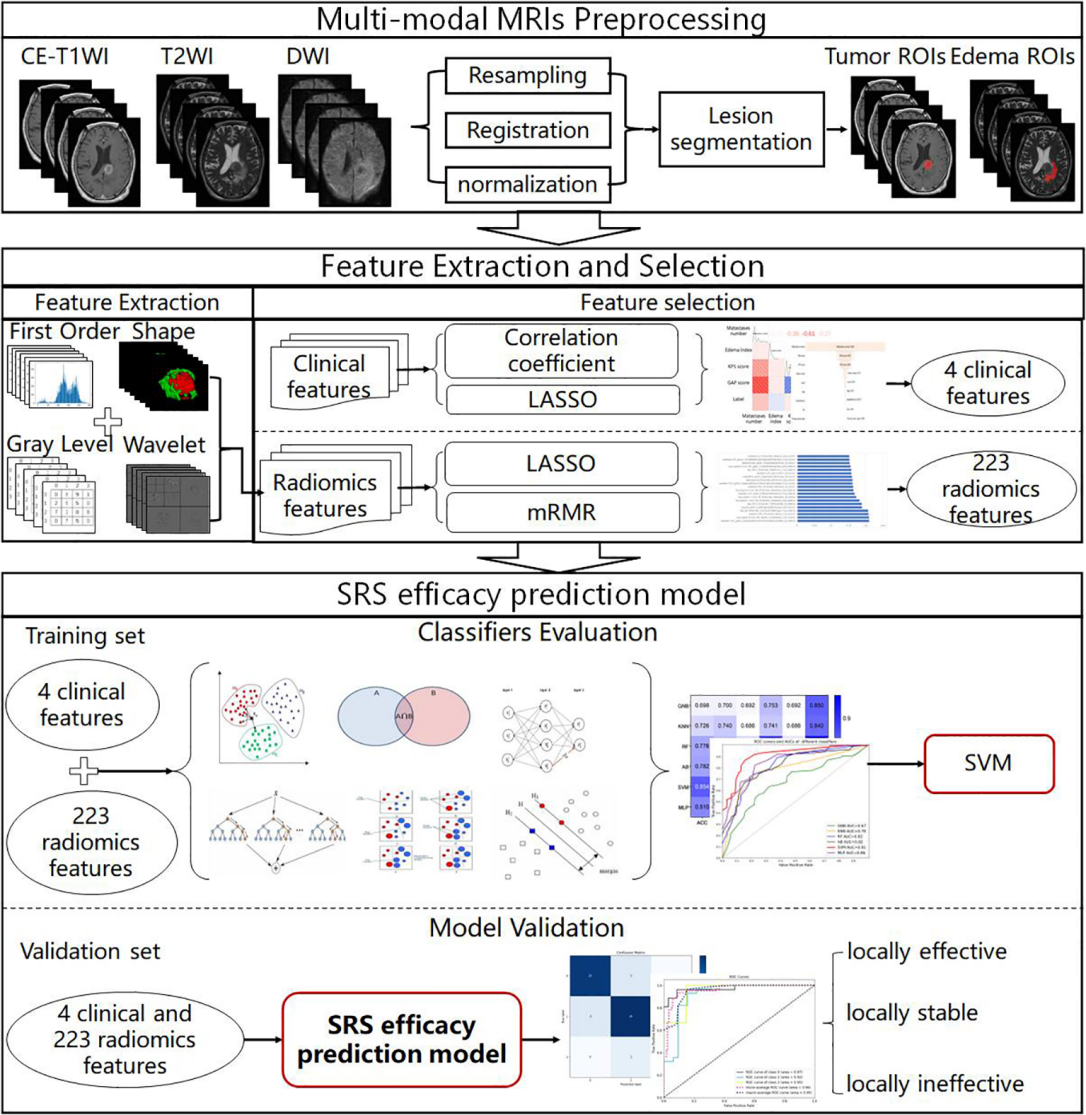
MRI data analysis flowchart.

The MRI data analysis flowchart is presented in **Figure 1**. We first performed alignment, resampling and normalization preprocessing of multimodal MRI images to obtain BM multimodal MRI images. Then, the importance of clinical features and radiomics features in the SRS efficacy prediction task was analyzed using feature selection methods (correlation coefficient, LASSO and mRMR filter), and 4 clinical features and 223 radiomics features with feature importance greater than zero and non-linear correlation were selected for modeling. Then, six classifiers were evaluated on the training set by a 5-fold cross-validation method, and the best performing SVM classifier was selected to construct the SRS efficacy prediction model. Finally, we evaluated the performance of the SRS efficacy prediction model proposed in this paper on the internal and external validation sets.

### Tumor region of interest delineation

To match the region of interest (ROI) to all MRI sequence images, we resampled all images to the same spacing (1×1×3 mm^3^) according to the Image Biomarker Standardization Initiative (IBSI) guidelines (29), using the linear difference resampling method in the SimpleITk package (Version 2.1.1.1, https://simpleitk.readthedocs.io/en/master/index.html). We used the Advanced Normalization Tools (ANTs) (https://github.com/ANTsX) to align all MRI image sequences to the CE-T1WI sequence. Then, the image intensities were normalized to 0-255 using the feature scaling method provided in SimpleITK.

### Tumor region of interest segmentation

Tumors were segmented by two radiologists (P.D. and H.L., with 16 years of experience in central nervous system radiology) blinded to diagnosis, using a semi-automatic tool ITK-SNAP (Version 4.0.0, http://www.itksnap.org/pmwiki/pmwiki.php), on CE-T1WI and T2WI axial images with 2 ROIs (masks): the whole tumor (enhancement and non-enhancement) and peritumoral edema. All masks were superimposed on three sequences (CE-T1WI, T2WI, and DWI).

### Radiomics feature selection

In this study, radiomics features were extracted in three dimensions using Pyradiomics (Version 3.0, https://pyradiomics.readthedocs.io/en/latest/features.html) on 6 combinations of 2 masks and 3 imaging sequences. Image transformations (including: wavelet, Laplacian of Gaussian (LoG), and square, square root, logarithm, and exponential filtes) were performed on the original images to obtain additional radiomics features. A total of 2153 features, including first order (First-Order, 414 features), shape-based (Shape, 14 features), Gray Level Cooccurrence Matrix (GLCM, 552 features), Gray Level Run Length Matrix (GLRLM, 368 features), Gray Level Size Zone Matrix (GLSZM, 368 features), Neighboring Gray Tone Difference Matrix (NGTDM, 115 features), and Gray Level Dependence Matrix (GLDM, 322 features), were extracted for each combination.

Given the size of the dataset, we extracted a sizable feature collection with a total of 12918 features (extracted from 3 sequences × 2 masks × 2153 features), which may lead to dimension explosion. Therefore, feature selection was necessary. Prior to feature selection, all features extracted from the two radiologists’ masks would be calculated by ICC (Intraclass correlation coefficient, a descriptive statistic used to measure the reproducibility of features), and only features with ICC greater than 0.9 would be included in feature selection and standardized steps. Then we used the LASSO filter to select the most important features from the features. Finally, considering features with highly linear correlations, we removed redundant features by using the mRMR filtering approach and retrieved the features for further work.

### Classifiers and evaluation indicators

Based on the performance of six classifiers, including Gaussian Naive Bayesian (GNB), k-Nearest Neighbors (KNN), random forest (RF), adaptive boosting (AB), support vector machine (SVM) with the linear kernel, and multilayer perceptron (MLP), all applied to the selected features, we chose the best model for SRS efficacy prediction.

In this study, we used the confusion matrix obtained by receiver operating characteristic (ROC) analysis to calculate the area under ROC curve (AUC), accuracy (ACC), positive predictive value (PPV), sensitive (SEN), specificity (SPE), and F1-score to assess individual model performance. The net reclassification improvement (NRI) metric was used to evaluate the significance of model improvement.

### Statistical analysis

We performed a statistical analysis of the results using IBM SPSS Statistics 26.0. Differences were considered statistically significant when the two-sided *P*<0.05. Moreover, the DeLong test was used in AUC difference test. In addition, feature selection, model construction and validation were performed using scikit-learn package (Version 1.0.2, https://scikit-learn.org/stable/) based on python (Version 3.10.7, https://www.python.org).

## Results

### The performance of the models in the training set

#### Model of the clinical features

**Figure 2:**
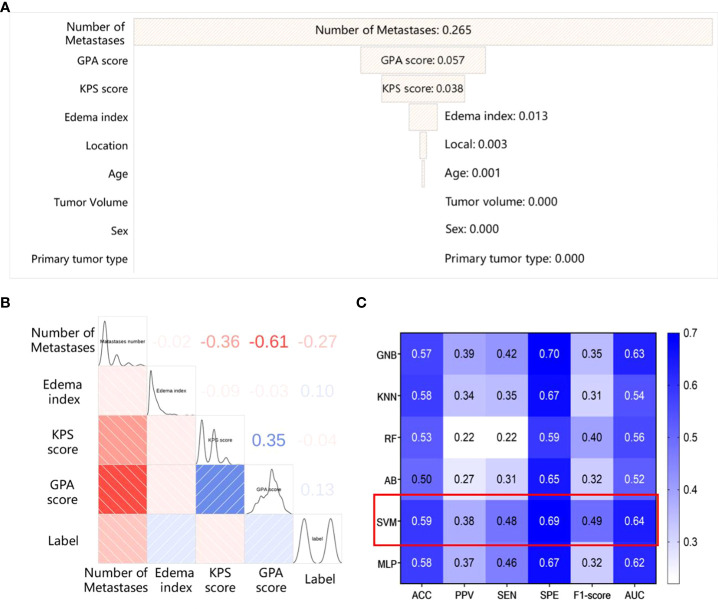
The results of the model based on clinical features **(A)** The importance of the clinical features; **(B)** The spearman correlation coefficient matrix between the selected clinical features; **(C)** The performance of different classifiers based on clinical features.

We compute Spearman correlation coefficient matrix and apply the logistic regression model to analyze the nine clinical characteristics of BM patients. The correlation and importance of the features are shown in **Figures 2A, B**. We find that the number of metastases and the Graded Prognostic Assessment (GPA) score are the most relevant clinical characteristics in the SRS efficacy prediction task, and the number of metastases also has the highest feature importance of 0.265. In addition, KPS score and edema index are also key clinical features for SRS efficacy prediction, while primary tumor type and sex are almost irrelevant for the task.

Hence, we use the above mentioned clinical features (the number of metastases, GPA score, edema index, KPS score) to establish a clinical feature-based SRS efficacy prediction model using different classifiers, and the results are shown in **Figure 2C**. Among the six classifiers considered, the SRS efficacy prediction model based on the SVM classifier yields the best performance (AUC=0.64, 95% CI: 0.62-0.66) on the training set.

#### Model of the radiomics features

**Figure 3:**
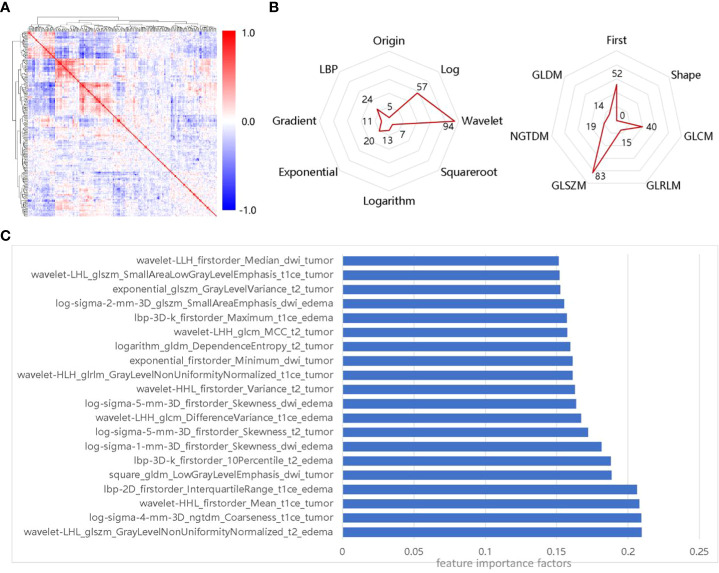
Importance of selected features, **(A)** Spearman correlation coefficient matrix for selected features; **(B)** Distribution of selected features in transformed images and feature classes; **(C)** Features of the top 10% of importance in the selected 223 radiomics features.

First, we select 223 radiomics features using the LASSO and mRMR filters. The distribution of feature Spearman correlation coefficients and feature categories are shown in **Figures 3A, B**. We find that GLSZM features account for the largest proportion of the feature class-based radar maps (37%), while wavelet features account for the largest proportion of image transformation-based radar maps (42%). In addition, we select features with feature importance in the top 10% for analysis, as shown in **Figure 3C**. Compared to multiple sequence images and ROIs, CE-T1WI-based features and tumor ROIs-based features are more significant for the SRS efficacy prediction task.

**Figure 4:**
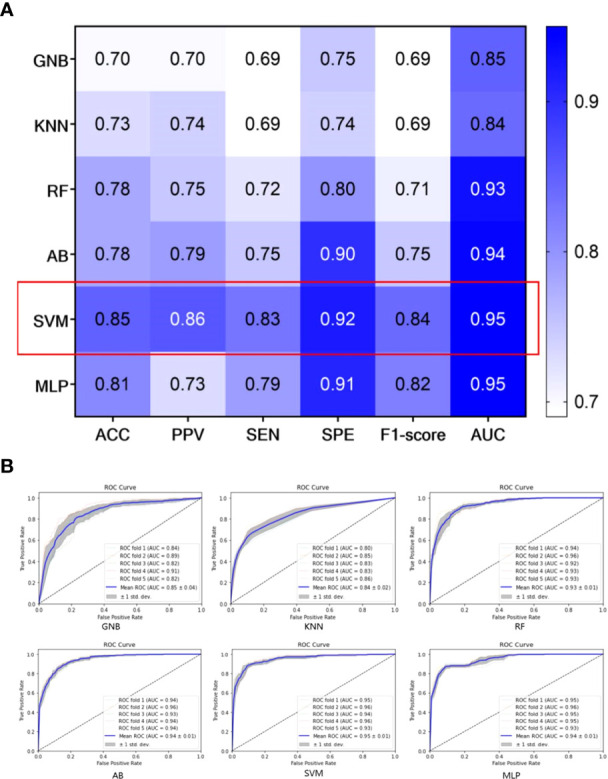
Performance of different classifiers on the training set; **(A)** The performance of different classifiers with 5-fold cross-validation; **(B)** macro-average ROC curves of different classifiers with 5-fold cross-validation.

To select the best classifier, we establish different SRS efficacy prediction models based on six classifiers using the 223 selected radiomics features and evaluate each of these classifiers using a 5-fold cross-validation. The results obtained using the six different classifiers on the training set are shown in **Figure 4A**. Among the six classifiers, the SVM classifier has the best performance in the SRS efficacy prediction model with ACC=0.85 (95% CI: 0.79-0.91), PPV=0.86 (95% CI: 0.82-0.90), SEN=0.83 (95% CI: 0.79-0.87), SPE=0.92 (95% CI: 0.90-0.94), and F1-score=0.84 (95% CI: 0.81-0.87). Then, the six classifiers are compared using the macro-average ROC curves with 5-fold cross-validation, which are shown in **Figure 4B**, and the SVM classifier still achieves the best AUC=0.95 (95% CI: 0.93-0.97).

### Differences in performance between radiomics and clinical models

Based on the above results, we establish the final SRS efficacy prediction model using four clinical features, 223 radiomics features, and the SVM classifier. In **Table 2**, we show that the final SRS efficacy prediction model demonstrates better classification performance compared with other models. The radiomics model significantly outperforms the clinical model with an improvement in the AUC value of 0.33 (training set: 0.95 vs. 0.62, *P*<0.05) and NRI value of 0.39. While the classification model based on clinical-radiomics features does not appear to provide significant improvement in the metrics considered compared with the radiomics model (training set: 0.95 vs. 0.95, *P*=0.15), and our final SRS efficacy prediction model established using SVM and clinical-radiomics features shows overall better classification performance with ACC=0.86 and AUC=0.95.

**Table 2:** The Performance of the models with clinical and radiomics features.

Classfier	Features		ACC	PPV	SEN	SPE	F1-score	AUC	NRI	
	clinical	radiomics								
SVM	✓		0.57	0.56	0.57	0.79	0.53	0.62	0.39	—
		✓	0.85	0.86	0.83	0.92	0.84	0.95		0.04
	✓	✓	0.86	0.85	0.86	0.93	0.85	0.95	—	

### The performance of the models in the validation sets

**Figure 5:**
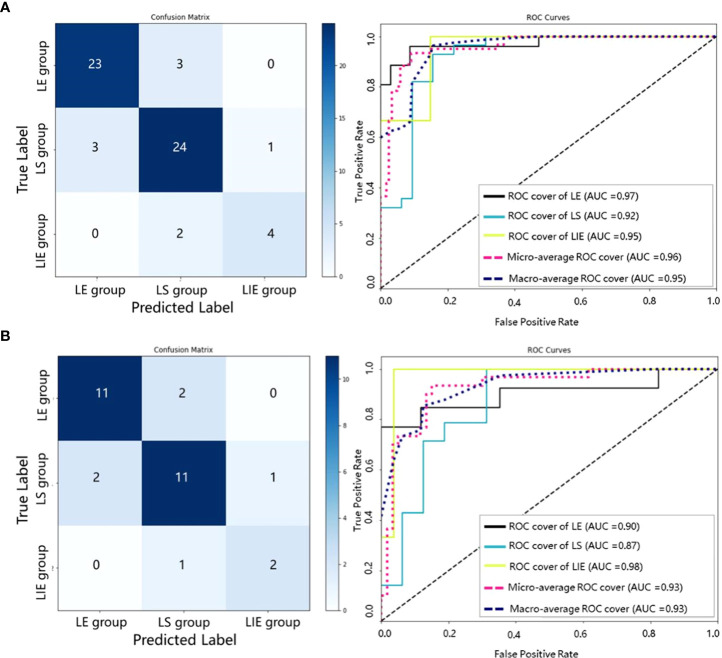
Confusion matrices and ROC curves of our SRS efficacy prediction models in the internal validation set **(A)** and the external validation set **(B)**.

In addition, we collect internal and external validation sets to evaluate the SRS efficacy prediction model established in this study. We emphasize that the validation sets are not used in any part of the model construction process – it is only used in the validation and evaluation of the final model. The results obtained from our model on the internal and external validation sets are shown in **Table 3** and **Figure 5**. The model achieves an AUC of more than 0.90 on both internal and external validation sets (internal validation AUC=0.95, and external validation AUC=0.93, respectively).

**Table 3:** Performance of SRS efficacy prediction models in internal and external validation sets.

Datasets	Models	ACC	PPV	SEN	SPE	F1-score	AUC
Internal-validation	radiomics-only	0.82	0.78	0.78	0.89	0.78	0.91
	clinical-radiomics	0.85	0.84	0.80	0.91	0.82	0.95
External-validation	radiomics-only	0.80	0.73	0.74	0.85	0.74	0.90
	clinical-radiomics	0.80	0.77	0.77	0.89	0.77	0.93

## Discussion

In this study, we develop a non-invasive ML prediction model integrating pre-treatment multimodal MRI radiomics features and relevant clinical risk factors to prospectively classify patients into LE, LS and LIE groups. Through validation, it indicates that this model is capable of outputting accurate predictions on both the training and validation sets. This may provide an auxiliary tool for precision diagnosis and treatment of BM patients.

In the study, we find that the number of metastases, GPA score, edema index, KPS score are significant in predicting the SRS efficacy of BM patients, which is similar to the findings of Baschnagel (30), Pontoriero (31), Park (32) and Noyama (33). The edema index, as an important clinical feature, is first used to assess the efficacy of SRS of BM, which was originally used for preoperative evaluation of peritumoral edema in meningiomas, to guide neurosurgeons in surgical planning (34). By analyzing the 223 radiomics features selected by the LASSO and mRMR filters, we find that 86 features are from CE-T1WI sequences (38.57%), and 125 features are from tumor ROIs (56.05%). In addition, texture features occupy the largest proportion (76.67%), and wavelet features also occupy a considerable proportion (42.15%). This implies that CE-T1WI sequence, tumor ROIs, advanced radiomics textures, and wavelet image features play an essential role in the task of predicting SRS efficacy.

We evaluate the performance of 18 combinations (6 classifiers × 3 feature combinations) for SRS efficacy prediction based on four clinical features and 223 radiomics features in the training set. The SVM classifier with a linear kernel achieves the best performance in all of the experiments. This is due to the ability of the SVM classifier to perform non-linear classification when applied to the selected features. According to this study, the radiomics models based on different classifiers outperforms the clinical models, which demonstrates the validity and effectiveness of the radiomics features. In addition, compared with the models based on clinical features or radiomics features alone, the model combining both the clinical and radiomics features demonstrates improved performance. Specifically, the AUC and ACC of the training set are improved by 0.01 and 0.02, respectively.

Our model has satisfactory performance on the validation sets, with the AUCs of both internal and external validation sets above 0.9. This also indicates that the generalizability of the proposed SRS efficacy prediction model. Moreover, it is found that our model has the highest AUC in the LIE group, as shown in **Figure 5**, which implies that our model can be particularly effective in identifying BM patients at risk of progression after receiving SRS therapy. The misclassified cases may be due to the relatively poor image quality, and the primary tumor types include lung cancer, colon cancer and breast cancer.

Existing research on BM indicates that there are many complicated factors influencing the efficacy of treatment (22, 35), such as the basic condition of patients, the histopathology type of primary tumors, the patients’ tolerance of chemotherapy and radiotherapy, the location, the number and the volume of BM, and the presence of extracranial metastases, etc. It is generally difficult to consider all the influence factors in one study. Thus, this study focuses on the patients with KPS≥70, no more than four intracranial metastases, no surgery or radiotherapy treatment before SRS therapy and no other concurrent therapies as the study objects. Besides, we note that the mechanism between radiotherapy and biological individuals are extremely complex (36). There are many uncontrollable factors and individual biological randomness influencing the prognosis of patients, which are not in the scope of this study.

The initial therapy of patients with BM mainly relies on local treatment (3), and the choice of initial treatment largely depends on the clinical experience and decision of neuro-oncology physicians – whether radiotherapy alone or radiotherapy after surgery. At present, surgery is only recommended for patients with a limited number of intracranial metastases (37). Moreover, surgery is an invasive treatment method with certain risks and complications. Hence, whether surgical intervention is required and when to use it is still a challenging issue that needs to be addressed comprehensively in neuro-oncology. The prediction model proposed in this study provides insights into this clinical decision from a quantitative perspective. Based on the predicting results of this model, once the patient is determined to be at risk of LF after SRS, the neuro-oncology physician can recommend surgery to remove the tumor, which is likely to alleviate the patient’s related neurological symptoms and improve the quality of life. Furthermore, tumor molecular markers and gene mutations obtained from surgical specimens can guide the drug selection of targeted therapy (2, 38). If it is determined that a patient is LE or LS, the patient can be treated based on the original treatment plan. For patients determined to be at risk of LF, surgery or SRS combined with WBRT may be recommended. The established ML model based on pre-treatment multimodal MRI radiomics and related clinical risk factors can prospectively and reliably predict the treatment response of patients with BM after receiving SRS therapy. To a certain extent, it can effectively assist neurologists and radiation oncologists in developing individualized therapeutic regimens and in turn minimizes the complications and adverse reactions of patients in the medical procedures.

RN is one of the main complications of radiotherapy for brain tumors (10), which usually occurs 6-9 months after radiotherapy (19, 39). RN and TP are remarkably similar in structural MRI features and clinical manifestations, yet they correspond to completely different treatments and prognosis (40). RN does not require treatment and will remain unchanged or gradually shrink over time. However, TP means treatment failure, requiring timely adjustments to treatment strategies. In order to ensure the objectivity and accuracy of the prediction and exclude the influence of RN on the prediction model, all patients who were determined to be LF in this study underwent perfusion weighted imaging (PWI) and magnetic resonance spectroscopy (MRS) subsequently. After comprehensive diagnosis by a multidisciplinary team consisting of experts from radiotherapy, neurosurgery, neuro-oncology, radiology, and pathology, the patients with questionable judgment results were excluded. Three patients were excluded due to questionable judgement results, and a total of 32 patients with progressive disease were ultimately enrolled in this study.

The following limitations exist in our study. First, the treatment response is evaluated using the pre-treatment MRI and the follow-up MRI approximately 60 days after treatment. Although it meets the evaluation requirements based on the RANO-BM criteria, it may only represent a short-term treatment response. The long-term efficacy analyses are still needed. Second, due to the inclusion of only patients with no more than four BM, the sample size of this study is limited. It is in the plan of our future work to conduct multicenter research, and explore the feasibility of including patients with more than four BM. Besides, more data from different scanner models and different image acquisition parameters need to be included in future studies to improve the generalization of the prediction model. Furthermore, in future studies, we will endeavor to incorporate genetic information of BM to increase the accuracy of prediction models.

## Conclusion

We present a novel non-invasive SRS treatment response prediction model of BM patients based on an ML approach. The model combines the pre-treatment multimodal MRI radiomics features and relevant clinical risk factors, and is capable of accurately identifying BM patients at risk of LF after SRS therapy. The proposed radiomics model shows excellent performances in both the internal and external validation sets, which can effectively aid in the development of an optimal treatment plan for patients in clinical applications.

## Data availability statement

The raw data supporting the conclusions of this article will be made available by the authors, without undue reservation.

## Ethics statement

The studies involving human participants were reviewed and approved by The Institutional Review Board of Huashan Hospital, Fudan University. Written informed consent for participation was not required for this study in accordance with the national legislation and the institutional requirements.

## Author contributions

PD, XL, and LS performed data acquisition and drafted the manuscript. LS, XW, and LC made substantial contributions to data acquisition. PD, XW, and LS were in charge of statistical analyses and data interpretation. PD, WX, and JC were responsible for recruiting patients. LS and AC made substantial contributions to the study design. PD, XL, and LS made substantial contributions to conception and design of the study. AC and DG provided professional guidance. PD, XL, and LS are the co-first authors of this article, and they contributed equally to this manuscript. AC and DG are the corresponding authors. All authors contributed to the article and approved the submitted version.
